# Influenza Vaccination and COVID-19 Mortality in the USA: An Ecological Study

**DOI:** 10.3390/vaccines9050427

**Published:** 2021-04-24

**Authors:** Claudio Zanettini, Mohamed Omar, Wikum Dinalankara, Eddie Luidy Imada, Elizabeth Colantuoni, Giovanni Parmigiani, Luigi Marchionni

**Affiliations:** 1 Department of Pathology and Laboratory Medicine, Weill Cornell Medicine, New York, NY 10065, USA; clz4002@med.cornell.edu (C.Z.); mao4005@med.cornell.edu (M.O.); wdd4001@med.cornell.edu (W.D.); eli4001@med.cornell.edu (E.L.I.); 2 Department of Biostatistics, Johns Hopkins University, Baltimore, MD 21205, USA; ejohnso2@jhmi.edu; 3 Department of Data Sciences, Dana-Farber Cancer Institute, Boston, MA 02215, USA; gp@jimmy.harvard.edu; 4 Department of Biostatistics, Harvard TH Chan School of Public Health, Boston, MA 02115, USA

**Keywords:** COVID-19, mortality, influenza, influenza vaccine, vaccination coverage

## Abstract

The COVID-19 mortality rate is higher in the elderly and in those with pre-existing chronic medical conditions. The elderly also suffer from increased morbidity and mortality from seasonal influenza infections; thus, an annual influenza vaccination is recommended for them. In this study, we explore a possible county-level association between influenza vaccination coverage in people aged 65 years and older and the number of deaths from COVID-19. To this end, we used COVID-19 data up to 14 December 2020 and US population health data at the county level. We fit quasi-Poisson regression models using influenza vaccination coverage in the elderly population as the independent variable and the COVID-19 mortality rate as the outcome variable. We adjusted for an array of potential confounders using different propensity score regression methods. Results show that, on the county level, influenza vaccination coverage in the elderly population is negatively associated with mortality from COVID-19, using different methodologies for confounding adjustment. These findings point to the need for studying the relationship between influenza vaccination and COVID-19 mortality at the individual level to investigate any underlying biological mechanisms.

## 1. Introduction

Over the past year, COVID-19 became a global health threat owing to its high rates of spread and mortality. By 7 April 2021, the total number of confirmed global cases reached more than 130 million, with over 2.5 million deaths [1]. Nearly 80% of symptomatic infections are mild [2], with the most frequent presentation including fever, fatigue, and a dry cough. Other symptoms also reported include, but are not limited to, sore throat, nausea, diarrhea, confusion, anosmia, and other taste abnormalities [3,4]. Nearly 14% of infected individuals develop severe disease with dyspnea and hypoxia. Critical illness is seen in about 5% of cases in the form of septic shock, and respiratory and multiorgan failure [2]. The risk of developing severe complications and mortality rates are higher in the elderly, males, and patients with comorbidities, especially those with hypertension, diabetes, and chronic respiratory conditions including asthma and chronic obstructive pulmonary disease (COPD) [5].

Viral coinfections, including influenza A and B, were reported in COVID-19 patients [6]. Seasonal influenza causes distinct outbreaks every year, with the attack rate varying in the range of 10%–20%. Similar to COVID-19, the morbidity and mortality rates of influenza are also higher in the elderly and in patients with chronic comorbidities; thus, routine annual vaccinations are highly recommended for these groups [7]. While the vaccine has a clear benefit for the elderly, the benefit is even higher for those with high-risk illnesses. The risk reduction with the vaccine is two- to fourfold higher in the elderly population with chronic underlying conditions compared to the healthy population of the same age group, even when there was a poor match between the vaccine and circulating strains [8], and when the vaccine effectiveness rate was as low as 10% [9]. Interestingly, Taksler et al. [10] found an inverse relationship between influenza vaccine coverage in adults aged 18–64 and influenza-related illnesses in those aged ≥65, which could be explained by reduced transmission in the community.

A plausible conjecture is that reducing the rates of chronic and acute respiratory comorbidities in high-risk populations could, in turn, lead to a reduction in the complications and deaths associated with COVID-19. On this basis, routine influenza vaccinations may be associated with lower morbidity and mortality rates from COVID-19 because immunized individuals have a lower rate of respiratory complications from influenza, and thus have a better baseline health condition to fight a COVID-19 infection. However, increased odds of non-SARS coronaviruses infections were recently reported among army personnel who received an influenza vaccination [11]. Therefore, influenza vaccination may increase the susceptibility to COVID-19 infection, and thereby be associated with increased COVID-19 morbidity and mortality through immune-mediated mechanisms such as antibody-dependent enhancement (ADE) [12].

Accordingly, we investigate whether influenza vaccination coverage in the elderly is related to COVID-19 mortality, and if so, in which direction. To this end, we analyze COVID-19 mortality rates and influenza vaccination coverage in the US at the county level up to 14 December 2020. In our analysis, we carefully adjusted for important social, economic, and health determinants to mitigate the intrinsic limitations of observational studies on the basis of aggregated data.

## 2. Materials and Methods

To evaluate the impact of influenza vaccination on COVID-19 mortality, we collected and analyzed data on vaccination coverage in the elderly (the number of people aged ≥65 who had received an influenza vaccine the previous season compared to the total population in the same age group), COVID-19 mortality, and other important socioeconomic, health-related, and environmental variables from the US. The cumulative numbers of COVID-19 cases and deaths until 14 December 2020 were considered, as this date was when the COVID-19 vaccination campaign started in the US.

### 2.1. Data Collection and Processing

The number of confirmed COVID-19 cases and deaths were obtained from the Johns Hopkins University (JHU) Center for Systems Science and Engineering (CSSE) COVID-19 repository [13], which aggregates and combines data reported by national and state sources. All of these sources include cases in which COVID-19 infection was laboratory-confirmed, and others in which it was considered the probable cause of death. At the time of our analysis, the JHU CSSE repository only reported deaths at the state level for the state of Rhode Island. Thus, the number of deaths in each county was obtained using the New York Times COVID-19 data repository (https://github.com/nytimes/covid-19-data, accessed on 22 April 2021). Information on influenza vaccination coverage in people ≥65 years old, medical conditions, and tobacco use (year, 2019) were obtained from the Center of Medicare Disparity Office of Minority Health (https://data.cms.gov/mapping-medicare-disparities, accessed on 22 April 2021).

Demographic data on household composition, gender, race, age, and poverty levels (year 2018) in each US county were retrieved from the Census Bureau (https://data.census.gov/cedsci/table?q=United%20States, accessed on 22 April 2021). The number of hospital beds per county (year 2020) was obtained from the Homeland Infrastructure Foundation (https://hifld-geoplatform.opendata.arcgis.com/datasets/hospitals/data?page=18, accessed on 22 April 2021). Lastly, information on the emissions of particulate matter (PM_2.5_; year, 2016) was originally reported by the Atmospheric Composition Analysis Group (http://fizz.phys.dal.ca/~atmos/martin/?page_id=140\V4.NA.02.MAPLE, accessed on 22 April 2021), and obtained and preprocessed by Wu et al. [14].

**Figure 1:**
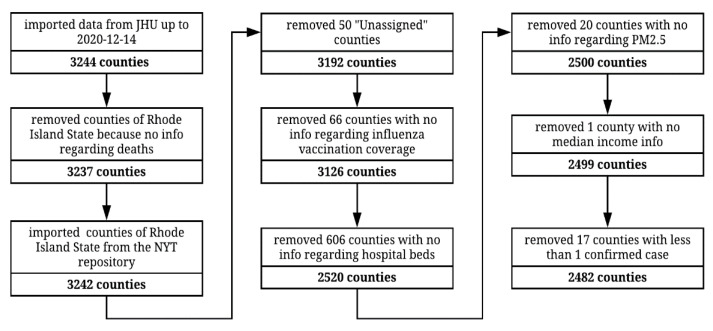
Steps of data collection and processing. Initial dataset comprised 3244 counties. COVID-19 data up to 14 December 2020 were imported from Johns Hopkins University repository. Since this repository does not report Rhode Island COVID-19-related deaths at the county level, that information was obtained from the New York Times repository instead. Counties with missing information regarding the variables of interest or confounders and those with zero cases were excluded from analysis. The final dataset used for the main analysis comprised 2482 counties.

Information was available for a total of 3244 counties across the 50 states and Washington DC, covering 22 January 2020 to 14 December 2020. Of these, 2482 counties were included in the final analysis since they had at least one confirmed COVID-19 case and no missing information for the other variables (as illustrated in Figure 1).

### 2.2. Confounding Adjustment with Generalized Propensity Score

Overall, the initial set of variables related to population demographics and clinical and territory characteristics was large (~300). We selected a set of potential confounders (40 variables) known to be related to either COVID-19 mortality (the outcome variable) alone or both COVID-19 mortality and influenza vaccination coverage (the exposure variable) [15]. These include underlying medical conditions [16,17], socioeconomic factors such as median income and education [18,19], environmental factors such as PM_2.5_ levels [14], and demographic factors such as age, sex, and race [20] (see Figure S1). We did not adjust for variables only related to exposure (influenza vaccination coverage) since they were not expected to confound the association under study [15].

To account for these potential confounders, we estimated a generalized propensity score (PS) for county-level influenza vaccination coverage in people ≥65 years old by regressing the logit of vaccination coverage on the confounding variables (Table S1). The propensity score is a balancing factor that corrects for the potential imbalance in confounding variables across values of the county-level influenza vaccination coverage, the exposure variable [15,21,22]. One advantage of using PS in a regression model—over accounting for all the confounding variables directly—is that it allows for summarizing the confounding variables into a single score [23], thus avoiding the threat of overfitting and increased sensitivity to model misspecification.

### 2.3. Modeling the Effect of Vaccine Coverage on COVID-19 Mortality

**Figure 2:**
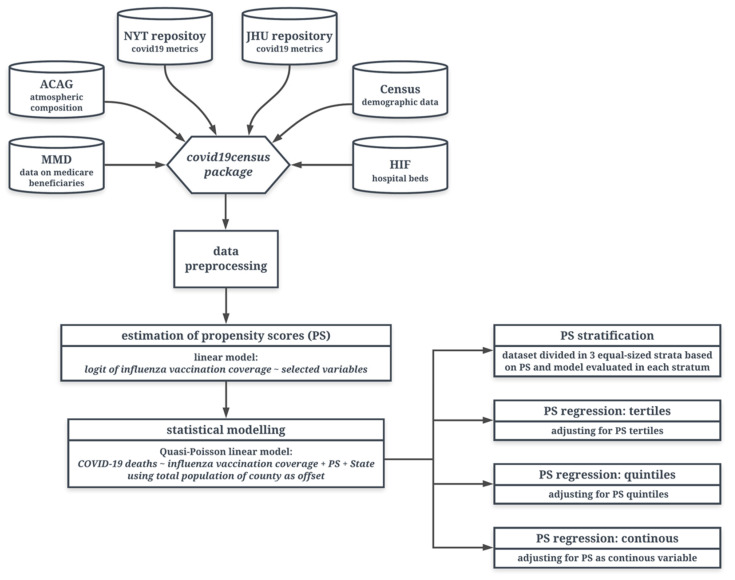
Steps used in this analysis, including data collection, preprocessing, and different propensity-score adjustment methods. COVID-19 data with other population and health characteristics were collected from six different sources and pooled into the COVID-19 census package. Counties meeting inclusion criteria were used in further analysis.

Since mortality-count data were overdispersed (mean = 118, SD = 426), we fit a quasi-Poisson regression to model the effect of influenza vaccination coverage in the elderly on the COVID-19 mortality rate [24]. We set up the county-level number of deaths from COVID-19 as the outcome, the influenza vaccination coverage in people ≥65 years old as the exposure variable, and the county population size as offset. We controlled for the potential confounders using different PS methods (see Figure 2). Lastly, we summarized the association by computing the mortality-rate ratio (MRR) of COVID-19 corresponding to a 10% increase in influenza vaccination coverage. We chose population mortality instead of case mortality to handle the effect of inconsistent COVID-19 testing policies across states.

In our main analysis, we used a combined PS stratification and regression adjustment approach to favor a balanced distribution of confounders and to handle the possible nonlinearity between the PS and the outcome. We divided the data into three equally sized strata on the basis of the estimated PS, and in each stratum, we fit a quasi-Poisson regression as described above, adding the PS as an additional covariate (estimate = mortality ~ vaccination coverage + PS + state + offset (population)). Lastly, we obtained an overall estimate of the association by applying the inverse-variance weighted method to the stratum-specific association estimates. Covariate balance was assessed by comparing the correlation between potential confounders and the exposure variable before and after stratification. Pearson’s correlation was calculated before dividing the dataset in strata and then again in each stratum. We then calculated the weighted average of the strata absolute correlations as an indicator of the independence between the confounders and influenza vaccination coverage. A reduction in the correlation between potential confounders and vaccination coverage after stratification would indicate an improved covariate balance [25,26].

Besides PS stratification, we employed other widely used PS approaches to adjust for the confounding variables to evaluate if the results were consistent with different methods. These included: (a) tertile PS: we divided the propensity score into tertiles, and included this categorical variable as a factor in the quasi-Poisson regression model; (b) quintile PS: we divided the PS into quintiles, and included it as a categorical variable in the model; and (c) continuous PS: we used the PS as a continuous variable (assuming linearity) [22]. For each PS regression method, we also included indicators for each US state in the main model to account for different testing and death-reporting policies together with other potentially hidden variables. A summary of our analytical approach is shown in Figure 2.

## 3. Results

### 3.1. Characteristics of Included Counties

A total of 2482 counties with at least one confirmed COVID-19 case were included in the analysis. In these counties, the median influenza vaccination coverage in people ≥ 65 years was 45% (interquartile range (IQR) = 13.8), and the median COVID-19 mortality rate (per 100,000 people) was 84.5 (IQR = 81.6). The overall distribution of the demographic, socioeconomic, and health-related variables is shown in Table S2.

### 3.2. Evaluating Covariates Balance before and after PS Stratification

**Figure 3:**
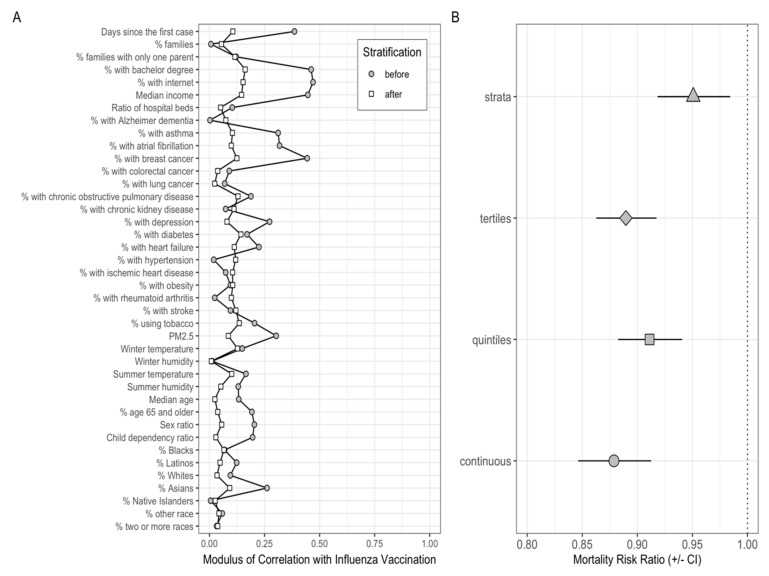
Effect of influenza vaccination coverage in the elderly on COVID-19 mortality after adjusting for the potential confounding variables. (**A**) Absolute correlation between potential confounders and influenza vaccination before (grey circles) and after (white squares) propensity-score (PS) stratification. (**B**) Mortality-rate ratio (MRR) of COVID-19 associated with a 10% increase in influenza vaccination coverage using different PS regression methods to adjust for confounding variables. Each symbol represents different PS regression methods: (a) strata: data divided into three strata on the basis of the estimated PS. In each stratum, we fit a quasi-Poisson regression model using the number of COVID-19 deaths as the outcome and influenza vaccination coverage in the elderly as the exposure variable, adjusting for the PS and using the total county population as offset; (b) tertiles: we divided the PS into tertiles and included it as a factor in the main model; (c) quintiles: same with quintiles; and (d) continuous: we used the PS as a continuous term in the outcome model. In all models, we also adjusted for US states. For stratified analyses, the MRR shown is the inverse-variance weighted MRR across the three strata.

In our main analysis, we divided these counties into three equally sized strata on the basis of the estimated PS. Each stratum included counties that share a similar distribution of the potential confounding variables (Table S2). Initially, there was high correlation between most of the confounding variables and influenza vaccination coverage, which was markedly reduced with PS stratification (Figure 3A).

### 3.3. Effect of Influenza Vaccination Coverage on COVID-19 Mortality Rates

Adjusted for a set of 40 potential confounders, the main models revealed a protective effect of influenza vaccination coverage in the elderly on the number of COVID-19 deaths at the county level. More specifically, a 10% increase in vaccination coverage among the elderly was associated with a 5% reduction in the number of deaths from COVID-19 using the PS stratification approach (inverse-variance weighted MRR: 0.95, 95% CI: 0.92–0.98). Similar results were obtained using tertile PS (MRR: 0.88, 95% CI: 0.86–0.92), quintile PS (MRR: 0.91, 95% CI: 0.88–0.94), and continuous PS (MRR: 0.88, 95% CI: 0.85–0.91) (as illustrated in Figure 3B)

When applying PS stratification analysis, we observed that the vaccination effect differed across the PS strata. In the first stratum, representing counties in the lowest PS tertile (Table S2), vaccination coverage was estimated to be associated with an increase in COVID-19 mortality rate (MRR: 1.10, 95% CI: 1.03–1.17). However, this association was reversed in the second (MRR: 0.90, 95% CI: 0.85–0.95) and third (MRR: 0.88, 95% CI: 0.82–0.94) strata. The inverse-variance weighted MRR across the three strata also indicated a negative association between influenza vaccination and COVID-19 mortality rates (see Figure 3B).

## 4. Discussion

In this study, we examine a possible connection between influenza vaccination in the elderly population and the risk of mortality from COVID-19. On the one hand, seasonal influenza infection is associated with the development of several respiratory complications, especially in the elderly [7]. On the other, a recent report pointed to the increased odds of non-SARS coronaviruses infections among army personnel who had received an influenza vaccination [11]. This observation was attributed by the authors on the possibility that vaccinated individuals lack the nonspecific immunity acquired by natural influenza infection, which would protect against infection by other viruses.

Public county-level data in the United States provided a basis to explore this issue. We quantified the county-level association between influenza vaccine coverage in people ≥65 years old and COVID-19-related mortality rates in these data. We focused on people ≥65 years old since they are more susceptible to influenza complications, and an annual influenza vaccination is thus recommended for them. This age group also has a higher mortality rate and a greater risk of developing severe complications from COVID-19 compared to the younger population [5]. In our analyses, we used different PS adjustment methods to control for a large set of potential confounding variables that might influence this association, including social and economic variables, education, chronic medical conditions, and other important demographic and environmental factors. Our results show that, at the county level, there is a reduction in the COVID-19 mortality rate associated with higher influenza vaccination coverage in the elderly population. This suggests a potential protective effect of influenza vaccination against COVID-19 mortality in the elderly and warrants further research to investigate the underlying mechanisms.

In our main analysis, we performed complete stratification of the data on the basis of the estimated PS. We noticed a heterogeneous effect of vaccination coverage across the three strata, with groups of counties with higher generalized PS (strata two and three) presenting a protective effect as opposed to counties in the first stratum. This heterogeneity of effect might be attributed to the different distribution of confounders since each stratum includes a group of counties with different characteristics from those in the other strata. For example, counties with the highest propensity of influenza vaccination (stratum three) tend to have, on average, better socioeconomic metrics, including higher rates of education and higher median income, compared with counties in the first stratum. Similarly, these counties tend to have a slightly smaller median age and lower percentage of people ≥ 65 years old. In terms of race, counties in the third stratum tend to have lower percentages of African American and Hispanic populations, and more White population compared to counties in the first stratum. The distribution of many health-related variables differs between the first and third strata, as counties in the third stratum tend to have a slightly higher percentage of people with asthma, atrial fibrillation, chronic kidney disease, depression, and obesity, and a lower percentage of people with diabetes, heart failure, and COPD. The different distribution of all these variables across the strata may account for the heterogeneity of the estimated effect and warrants further individual-level studies to explore the effect of these variables on influenza vaccine administration and COVID-19 outcomes. Nonetheless, the inverse-variance weighted MRR across the three strata reveals a negative association between vaccination coverage in the elderly and COVID-19 mortality, which is consistent with the effect estimated by the other PS regression methods.

Our results are consistent with those reported by other studies at the regional [27,28] and individual levels. One study examined the association between influenza vaccination and COVID-19 outcomes in hospitalized patients with COVID-19 in Brazil, and found a protective effect with a recent vaccination against severe outcomes including ICU admission and mortality [29]. Similarly, two other studies found a negative association between influenza vaccination and severe COVID-19 outcomes [30,31]; however, another study found that the vaccine does not have a significant effect on COVID-19 hospitalization and mortality except in those ≥65 years old [32]. Other reports showed a negative link between influenza vaccination and SARS-CoV-2 seropositivity [33,34].

Our results may be explained by the vaccine effect on reducing the rate of hospitalization and severe cardiopulmonary complications in the elderly. Evidence suggests that the vaccine benefit is high in the elderly, especially for those with pre-existing chronic lung diseases and other high-risk conditions [8]. Furthermore, influenza vaccination was found to have a protective effect against cardiovascular events [35], confirming previous findings that linked influenza infection to increased rates of hospitalization and death from myocardial infarction [36,37]. This, in turn, suggests that counties with higher influenza vaccination coverage in the elderly might have less saturated healthcare systems, which translates to lower rates of COVID-19 mortality compared to counties with lower vaccination coverage.

A complementary explanation for a putative protective effect is that unvaccinated elderly individuals are at risk of persistent viral infections, leading to a decline in T-cell diversity, which, in turn, impairs the immune response against other pathogens, including SARS-CoV-2 [38,39]. Influenza vaccination, on the other hand, does not induce a strong, virus-specific CD8 T-cell immune response, as seen with natural infection [40]. While this is considered to be a major disadvantage of inactivated influenza vaccines, it may be beneficial in clearing SARS-CoV-2 infection, as vaccinated individuals have more T-cell diversity (and thus, better chances to fight other viruses) compared to those with a natural influenza infection. Importantly, the influenza virus was shown to induce apoptosis and impair the cytotoxic effect of natural killer (NK) cells [41], ultimately impairing the host immune defense mechanisms against other pathogens, potentially including SARS-CoV-2, especially in the acute phase of the disease. Additionally, consistently unvaccinated individuals are more likely to have a higher proportion of influenza-specific resident memory T cells (T*_RM_*) in their lungs, which are highly proliferative and highly productive of inflammatory cytokines [42]. This, in turn, might contribute to the exaggerated inflammatory response and severe acute respiratory distress syndrome (ARDS) seen in some COVID-19 patients [43]. Similarly, a hyperinflammatory response was also seen in some children following COVID-19 infection, a condition called multisystem inflammatory syndrome in children (MIS-C) [44]. The pathophysiology of MIS-C is still unclear, but it is suggested to be an abnormal and delayed immune response to SARS-CoV2 infection characterized by increased activation of memory T cells accompanied by highly elevated inflammatory markers and persistent cytopenia [45]. MIS-C might be linked to the high proportion of airway T_RM_ resulting from viral respiratory tract infections, including respiratory syncytial virus (RSV) and influenza [46]; however, further studies are needed to investigate this association and to examine the role of vaccination against influenza and other respiratory pathogens in protecting against MIS-C. Furthermore, the influenza A virus was recently shown to upregulate ACE2 receptors in lung alveolar cells [46], suggesting that a recent influenza infection could lead to more severe pulmonary complications from COVID-19 since these same receptors are used by SARS-CoV-2 for cellular entry.

We regard our work as a preliminary study, and acknowledge several limitations. While our analysis considered many of the potential confounding factors that may affect the association under study, several important confounders were unmeasured in our data, including the quality of healthcare systems, the degree of compliance with preventive measures, and the difference in death-reporting policies between states. Testing availability and recommendations, especially in the initial phase of the pandemic, varied between states. We mitigated this issue by using the total county population as the offset in our models rather than the total number of confirmed cases. We were unable to restrict the outcome variable (COVID-19 mortality) to the same age group of the exposure variable (influenza vaccination coverage in ≥65-year-olds) because available county-level COVID-19 statistics are not stratified by age. However, the difference in the denominator of these two variables (all populations for COVID-19 mortality and elderly populations for vaccination coverage) is unlikely to affect the results of this analysis because most COVID-19 deaths in the US are among the elderly [47]. Lastly, based on the available information, it is still unclear if the negative association with COVID-19 mortality could also be observed with natural influenza infection or with other viral vaccines.

In conclusion, our analysis showed a negative association between influenza vaccination coverage in the elderly and COVID-19 mortality at the US county level. This effect was also reported by other studies at the individual level, warranting further research to investigate if there are any underlying biological mechanisms.

## Data Availability

All data used in this study, along with detailed information about data sources, are available in the form of the R package covid19census (dev2019 branch), freely distributed from GitHub (https://github.com/c1au6i0/covid19census/tree/dev2019 (accessed on 23 April 2021)). All scripts and code used to preprocess and analyze the data are available from https://github.com/marchionniLab/covid19influenza (accessed on 23 April 2021).

## Supplementary Materials

The following are available online at https://www.mdpi.com/article/10.3390/vaccines9050427/s1: Figure S1: Correlation matrix of the potential confounders, mortality, and case rates. Highly correlated variables are clustered together. Table S1: Propensity of influenza vaccination based on confounding variables. Table S2: Mean distribution of COVID-19-related metrics, influenza vaccination coverage, and potential confounding variables in each stratum.
