# Supplementary material for: Influenza Vaccination and COVID-19 Mortality in the USA: An Ecological Study
Source: Vaccines (Basel). 2021 Apr 24;9(5):427. doi: 10.3390/vaccines9050427 (PMC8145634; doi:10.3390/vaccines9050427)
Supplement: Supplementary file 1 [file vaccines-09-00427-s001.zip › vaccines-1179756-supplementary.pdf]

Table S1. The propensity of influenza vaccination based on the confounding variables. The propensity score was calculated by regressing the logit of influenza vaccination coverage on the potential confounding variables.

Term	Estimate	SD	Statistic	*p*.value
Intercept	−0.83	1.20	−0.69	0.49
% families	0.52	0.22	2.37	0.02
% families with only one parent	0.19	0.32	0.60	0.55
% with bachelor degree	1.19	0.27	4.37	< 0.01
% with internet	0.07	0.15	0.47	0.64
Ratio of hospital beds	−0.71	1.66	−0.43	0.67
% with Alzheimer dementia	−1.25	0.44	−2.85	< 0.01
% with asthma	3.22	0.76	4.25	< 0.01
% with atrial fibrillation	2.14	0.68	3.17	< 0.01
% with breast cancer	6.40	1.35	4.73	< 0.01
% with colorectal cancer	−3.59	1.84	−1.95	0.05
% with lung cancer	−0.59	2.22	−0.27	0.79
% with chronic obstructive pulmonary disease	−1.43	0.39	−3.63	< 0.01
% with chronic kidney disease	1.36	0.26	5.12	< 0.01
% with depression	1.79	0.31	5.82	< 0.01
% with diabetes	−1.60	0.34	−4.71	< 0.01
% with heart failure	−0.80	0.36	−2.24	0.03
% with hypertension	0.89	0.23	3.82	< 0.01
% with ischemic heart disease	−0.10	0.23	−0.45	0.65
% with obesity	−0.31	0.13	−2.31	0.02
% with rheumatoid arthritis	−0.54	0.21	−2.61	0.01
% with stroke	2.05	0.97	2.11	0.03
% using tobacco	−1.41	0.38	−3.67	< 0.01
Median income	0.00	0.00	1.28	0.20
$PM_{2.5}$	0.03	0.00	6.70	< 0.01
Summer temperature	0.01	0.00	1.43	0.15
Summer humidity (%)	0.00	0.00	1.79	0.07
Winter temperature (K)	−0.01	0.00	−4.36	< 0.01
Winter humidity (%)	0.00	0.00	−0.72	0.47
% age 65 and older	0.66	0.55	1.20	0.23
Median age	−0.02	0.00	−3.54	< 0.01
Sex ratio	0.00	0.00	−3.76	< 0.01
Child dependency ratio	−0.01	0.00	−4.59	< 0.01
% African Americans	−0.18	0.15	−1.19	0.23
% Latinos	−0.15	0.10	−1.48	0.14
% Whites	0.34	0.16	2.06	0.04
% Asians	−1.23	0.41	−3.03	< 0.01
% Native Islanders	3.93	4.92	0.80	0.42
% other race	0.25	0.29	0.87	0.39
% two or more races	0.20	0.52	0.38	0.70
Days since the first case	0.00	0.00	8.05	< 0.01

SD: standard deviation.

Table S2. Mean distribution of COVID−19 related metrics, influenza vaccination coverage and potential confounding variables overall and in each stratum. A total of 2482 counties were included in the analysis and were divided into three equal−sized strata based on the estimated PS.

			PS strata		
**Category**	**Variable**	**Overall**	**Stratum 1**	**Stratum 2**	**Stratum 3**
	Number of counties	2482	828	827	827
**COVID−19**	Death rate (per 100,000 people)	99.04 (75.04)	115.99 (91.13)	101.09 (74.63)	80.03 (48.73)
	Confirmed−case rate (per 100,000 people)	5786.21 (2474.45)	6136.41 (2576.02)	5920.19 (2533.65)	5301.62 (2225.21)
	Number of days since first case	259.75 (22.53)	246.06 (31.28)	262.38 (12.21)	270.83 (8.87)
**Socioeconomic factors**	% families	0.66 (0.05)	0.66 (0.05)	0.67 (0.04)	0.66 (0.06)
	% families with only 1 parent	0.16 (0.05)	0.17 (0.06)	0.16 (0.04)	0.15 (0.03)
	% with bachelor or higher degree	0.14 (0.06)	0.11 (0.04)	0.13 (0.04)	0.19 (0.06)
	% with internet	0.74 (0.09)	0.67 (0.09)	0.73 (0.06)	0.80 (0.06)
	Median income ($1,000)	51.91 (13.55)	43.79 (9.35)	50.07 (8.81)	61.88 (14.75)
**Health factors***	% influenza vaccination coverage	0.43 (0.10)	0.35 (0.09)	0.44 (0.08)	0.52 (0.06)
	Ratio of hospital beds	3.68 (4.41)	4.54 (5.90)	3.38 (3.45)	3.11 (3.22)
	% with Alzheimer's disease	0.10 (0.02)	0.10 (0.03)	0.10 (0.02)	0.10 (0.02)
	% with asthma	0.04 (0.01)	0.04 (0.01)	0.04 (0.01)	0.05 (0.01)
	% with atrial fibrillation	0.08 (0.02)	0.08 (0.02)	0.09 (0.01)	0.09 (0.01)
	% with breast cancer	0.03 (0.01)	0.03 (0.01)	0.03 (0.01)	0.03 (0.01)
	% with colorectal cancer	0.01 (0.004)	0.01 (0.004)	0.01 (0.004)	0.01 (0.003)
	% with lung cancer	0.01 (0.003)	0.01 (0.004)	0.01 (0.003)	0.01 (0.003)
	% with obstructive pulmonary disease	0.13 (0.03)	0.13 (0.04)	0.13 (0.03)	0.11 (0.03)
	% with chronic kidney disease				
	% with depression	0.24 (0.05)	0.24 (0.05)	0.25 (0.05)	0.25 (0.04)
	% with diabetes	0.19 (0.03)	0.17 (0.04)	0.19 (0.03)	0.20 (0.03)
	% with heart failure	0.27 (0.05)	0.28 (0.06)	0.27 (0.05)	0.26 (0.04)
	% with hypertension	0.15 (0.03)	0.16 (0.04)	0.15 (0.03)	0.14 (0.02)
	% with ischemic	0.58 (0.08)	0.57 (0.10)	0.58 (0.08)	0.57 (0.07)
	heart disease	0.27 (0.05)	0.28 (0.06)	0.28 (0.05)	0.26 (0.04)
	% with obesity	0.21 (0.07)	0.20 (0.07)	0.22 (0.07)	0.22 (0.06)
	% with rheumatoid	0.34 (0.05)	0.34 (0.06)	0.34 (0.05)	0.34 (0.04)
	arthritis	0.03 (0.01)	0.03 (0.01)	0.03 (0.01)	0.04 (0.01)
	% stroke or transient ischemic attack				
	% using tobacco	0.11 (0.03)	0.12 (0.04)	0.12 (0.03)	0.10 (0.03)
**Environmental factors**	Average PM2.5 (microg/m3)	8.51 (2.50)	7.27 (2.55)	8.58 (2.25)	9.68 (2.08)
	Winter temperature (K)	280.35 (6.69)	282.04 (6.61)	280.04 (6.82)	278.96 (6.26)
	Winter humidity (%)	87.54 (4.89)	87.27 (5.00)	88.25 (4.78)	87.10 (4.81)
	Summer temperature (K)	303.09 (3.20)	304.01 (3.39)	303.03 (3.23)	302.25 (2.71)
	% summer humidity	89.02 (9.35)	86.85 (11.33)	90.06 (8.92)	90.16 (6.87)
	Median age	40.80 (5.13)	41.68 (5.58)	41.17 (4.59)	39.53 (4.93)

**Population demographics**	% 65 years or more of age	0.18 (0.04)	0.19 (0.05)	0.18 (0.04)	0.17 (0.04)
	Sex ratio	99.96 (9.71)	103.11 (14.01)	99.38 (7.13)	97.39 (4.39)
	Child dependency ratio	37.97 (6.23)	39.90 (7.33)	38.10 (5.05)	35.92 (5.42)
**Race**	% African Americans	0.09 (0.14)	0.12 (0.19)	0.08 (0.13)	0.08 (0.10)
	% Latinos	0.10 (0.14)	0.13 (0.18)	0.08 (0.12)	0.08 (0.08)
	% Whites	0.83 (0.16)	0.80 (0.20)	0.85 (0.14)	0.84 (0.12)
	% Asians	0.02 (0.03)	0.01 (0.01)	0.01 (0.03)	0.03 (0.03)
	% Native Islanders	0.001 (0.002)	0.001 (0.001)	0.001 (0.002)	0.001 (0.001)
	% other race	0.02 (0.04)	0.03 (0.05)	0.02 (0.04)	0.02 (0.03)
	% two more races	0.02 (0.02)	0.02 (0.02)	0.02 (0.02)	0.03 0.01)

*Health factors refers to those ≥ 65 years old only. Standard deviations are in parenthesis.

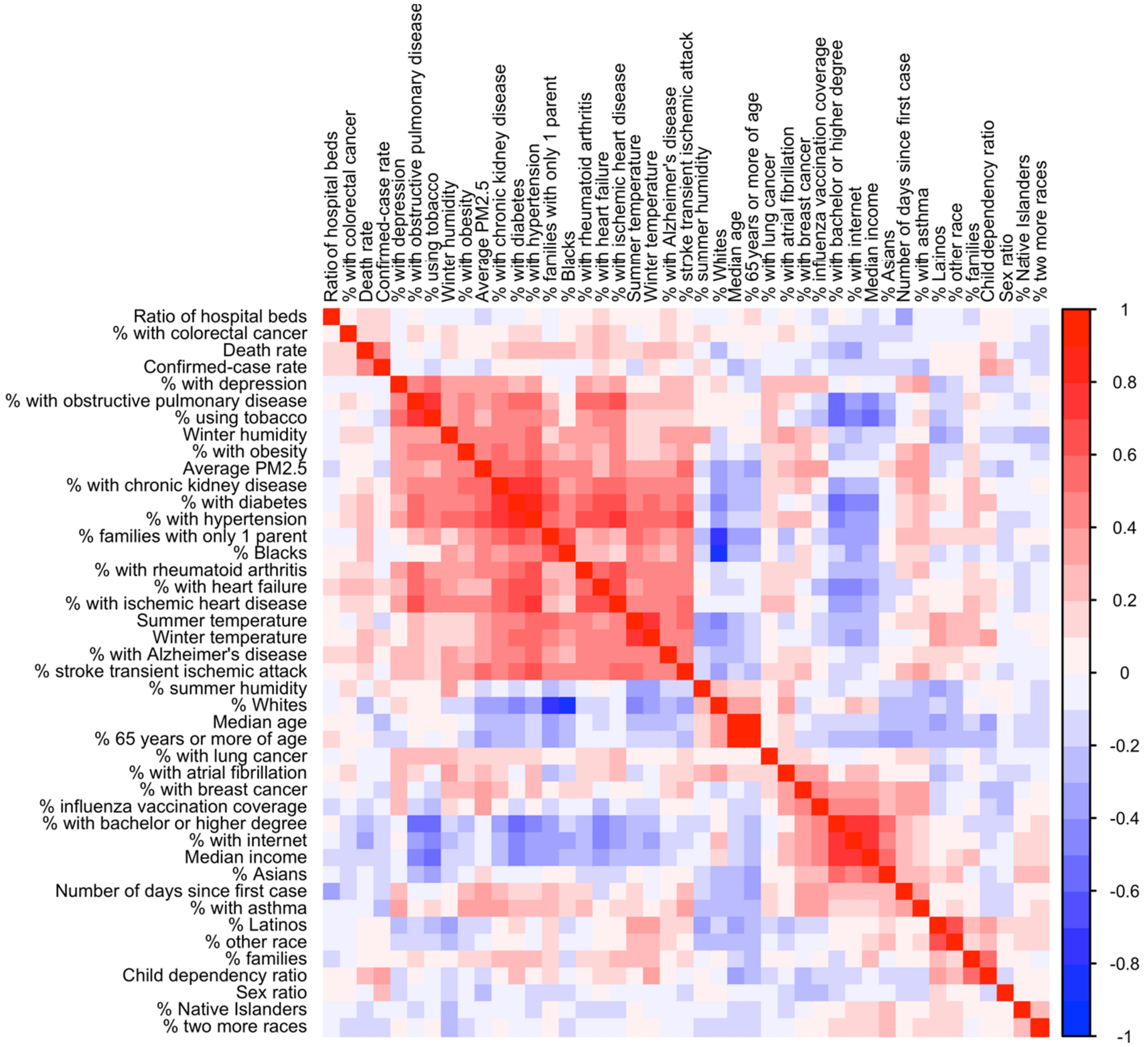

Figure S1. Correlation matrix of the potential confounders, mortality and case rates. Highly correlated variables are clustered together.
